# Associations of Arachidonic Acid Synthesis with Cardiovascular Risk Factors and Relation to Ischemic Heart Disease and Stroke: A Univariable and Multivariable Mendelian Randomization Study

**DOI:** 10.3390/nu13051489

**Published:** 2021-04-28

**Authors:** Ting Zhang, Shiu-Lun Au Yeung, C. Mary Schooling

**Affiliations:** 1 School of Public Health, Li Ka Shing Faculty of Medicine, The University of Hong Kong, Hong Kong, China; zhangt77@connect.hku.hk (T.Z.); ryanaysl@connect.hku.hk (S.-L.A.Y.); 2 School of Public Health and Health Policy, City University of New York, New York, NY 10027, USA

**Keywords:** arachidonic acid, cardiovascular disease, coagulation, inflammation, lipid, mendelian randomization

## Abstract

Arachidonic acid (AA), a major long-chain omega-6 polyunsaturated fatty acid, is associated with ischemic heart disease (IHD) and stroke. We assessed bi-directional associations of AA synthesis reflected by plasma phospholipid AA with CVD risk factors, and identified mediators of associations of AA with IHD and stroke using Mendelian randomization (MR). We used two-sample MR to assess bi-directional associations of AA synthesis with lipids, blood pressure, adiposity, and markers of inflammation and coagulation. We used multivariable MR to assess mediators of associations of AA with IHD and stroke. Genetically predicted AA (% of total fatty acids increase) was positively associated with apolipoprotein B (ApoB, 0.022 standard deviations (SD), 95% confidence interval (CI) 0.010, 0.034), high-density (0.030 SD, 95% CI 0.012, 0.049) and low-density lipoprotein cholesterol (LDL-C, 0.016 SD, 95% CI 0.004, 0.027) and lower triglycerides (−0.031 SD, 95% CI −0.049, −0.012) but not with other traits. Genetically predicted these traits gave no association with AA. The association of AA with IHD was attenuated adjusting for ApoB or LDL-C. Genetically predicted AA was associated with lipids but not other traits. Given ApoB is thought to be the key lipid in IHD, the association of AA with IHD is likely mediated by ApoB.

## 1. Introduction

Dietary fats have long been considered to affect cardiovascular disease (CVD) [1], with dietary guidelines recommending substituting polyunsaturated fatty acids (PUFA) for saturated fatty acids to prevent CVD [2,3]. However, the effects of specific PUFAs on CVD remain controversial. Of particular interest is the potential benefits of marine long-chain omega-3 PUFAs [3], with purified eicosapentaenoic acid (EPA) ethyl ester showing cardiovascular benefits [4] but EPA plus docosahexaenoic acid (DHA) showing little effect [5]. In contrast, concern has arisen about the potential harm of arachidonic acid (AA), an important long-chain omega-6 PUFA derived from dietary intake of animal foods (e.g., meat, egg, and liver) [6], possibly because AA competes with EPA for cyclooxygenase and lipoxygenase to form eicosanoids with potential effects on inflammatory and coagulation pathways [7]. Recently, Mendelian randomization (MR) studies, using genetic instruments to predict plasma phospholipid AA, have suggested positive effects on ischemic heart disease (IHD) and ischemic stroke [8,9,10]. Whether AA affects CVD risk factors, such as lipid profile, blood pressure, adiposity, inflammation and coagulation, and whether any of them mediates the relation of AA with IHD and ischemic stroke is unknown.

Observationally, circulating AA is positively associated with low-density lipoprotein cholesterol (LDL-C) and inversely associated with triglycerides (TG) [11], interleukin (IL)-6 and IL-1 receptor antagonist (IL-1Ra) [12]. Randomized controlled trials (RCTs) suggest dietary or supplemental AA has little effect on lipid profile [13,14,15], blood pressure [14,16], body composition [14,17], tumor necrosis factor-α (TNF-α) or CRP [13,16] and inconsistent effects on IL-6 [13,16,17] and platelet aggregation and coagulation [14,16,18,19,20] but are circumscribed by short duration, small samples and interpretation depending on the choice of control. Most of the RCTs were conducted in men only [13,14,15,17,18,20] and no trial has examined sex differences, although vulnerability to CVD differs by sex.

MR may provide estimates of lifelong effects of AA with minimal confounding. A previous MR study found plasma AA increased LDL-C and HDL-C and lowered diastolic blood pressure (DBP) [10]. The study used genetic variants from the *FADS1* and *FADS2* genes, including rs174547, which was homozygous in the archaic Flores people and is homozygous in Inuit people today [21], which encode key desaturases in PUFA synthesis and affect several fatty acids and traits [9], precluding the isolation of the effects of AA. No MR study has examined the associations of AA with apolipoprotein B (ApoB), an increasingly recognized predominant lipid trait in IHD [22] and ischemic stroke [23], adiposity, inflammation or coagulation, or examined sex differences. Using genetic instruments predicting AA synthesis, reflected by plasma phospholipid AA, independent of the Flores and Inuit haplotype [24], we conducted univariable MR to assess the associations of AA synthesis with lipid profile, blood pressure, adiposity, and markers of inflammation and coagulation, overall and by sex. We checked whether any of these factors affected AA synthesis. Multivariable MR was used to assess potential mediators of AA synthesis on IHD and ischemic stroke.

## 2. Materials and Methods

### 2.1. Genetic Instruments for AA Synthesis

Genetic variants, i.e., single-nucleotide polymorphisms (SNPs), associated with plasma phospholipid AA (% of total fatty acids) at genome-wide significance (*p* < 5 × 10^−8^) were identified and genetic association with AA were obtained from summary statistics provided by the Cohorts for Heart and Aging Research in Genomic Epidemiology (CHARGE) Consortium comprising 8631 individuals of European descent adjusted for age, sex, site, and 2~10 principal components [24]. SNPs available for all cohorts in the GWAS with minor allele frequency >1% were selected, and independent SNPs (*r*^2^ < 0.01) identified using the “ld_clump” function from MR-Base. To ensure the variants for AA synthesis were independent of the Flores and Inuit haplotype (tagged by rs174547), which not only affects AA but also affects other fatty acids and traits, we used two sets of instruments, one affecting AA synthesis independent of rs174547 (*r*^2^ < 0.01), and the other affecting AA synthesis only through rs174547.

SNPs with pleiotropic effects (*p* < 5 × 10^−8^) in any of three comprehensive curated genetic cross-references, i.e., Ensembl 102 (http://www.ensembl.org/index.html, accessed 20 January 2021), PhenoScanner (www.phenoscanner.medschl.cam.ac.uk, accessed 20 January 2021) and the MRC Integrative Epidemiology Unit (IEU) GWAS database (https://gwas.mrcieu.ac.uk, accessed 20 January 2021) were excluded. We also excluded SNPs associated with key confounders (education, Townsend index, smoking, alcohol drinking, and physical activity) of the AA synthesis outcome association in the UK Biobank (UKB) [25] (*p* < 5 × 10^−8^). We excluded SNPs more strongly associated with other omega-6 PUFA in sensitivity analysis.

### 2.2. Genetic Associations with Risk Factors for CVD

Genetic associations with lipid profile, including circulating non-fasting LDL-C, TG, HDL-C, ApoB, and apolipoprotein A-I (ApoA-I), and systolic blood pressure (SBP) and DBP were obtained from the UK Biobank [25] pan-ancestry summary statistics (https://pan.ukbb.broadinstitute.org/, released 16 June 2020, assessed 15 January 2021) encompassing 420,531 people of European ancestry only. We also obtained summary genetic associations with fasting LDL-C, TG, and HDL-C from the Global Lipids Genetics Consortium (GLGC) in up to 188,578 people of European ancestry only [26]. Genetic associations with BMI in 681,275 individuals of European ancestry were obtained from the Genetic Investigation of ANthropometric Traits (GIANT) Consortium and the UK Biobank meta-analysis [27]. Genetic associations with circulating IL-1β, IL-1Ra, IL-6 and TNF-α were obtained from a GWAS of cytokines in 8293 Finnish individuals unadjusted for BMI [28,29]. Genetic associations with circulating soluble IL-6 receptor (sIL-6R) and C-reactive protein (CRP) were obtained from the INTERVAL study in 3301 individuals of European ancestry [30] and the CHARGE Inflammation Working Group (CIWG) in 204,402 European individuals [31], respectively. Genetic associations with markers of coagulation, specifically activated partial thromboplastin time (APTT), prothrombin time (PT), and circulating fibrinogen, in 58,110 Japanese individuals without anticoagulant therapy or cirrhosis, were taken from Biobank Japan [32]. We did not use genetic associations with coagulation from a European population because they were not comprehensively available. Sex-specific genetic associations with lipids, blood pressure, BMI, and CRP were obtained from the UK Biobank [25] (http://www.nealelab.is/uk-biobank/, released 31 July 2018, assessed 2 March 2021) including 167,020 white British men and 194,174 women. The details of each GWAS are summarized in Table S1.

For multivariable MR analyses and MR analysis of CVD risk factors on AA synthesis, we used published genetic instruments for LDL-C (220), TG (440), ApoB (255), HDL-C (534), and ApoA-I (440) [22], SBP (130), DBP (91) [33], BMI (97) [34], IL-1β (1) [35], IL-1Ra (2) [36], IL-6 (3) [37], IL-6R (1) [38], CRP (52) [31], APTT (8), PT (2) [39] and fibrinogen (41) [40] at genome-wide significance from existing GWAS in individuals of mainly European ancestry (Table S2). We used the MR-Base “ld_clump” function to obtain independent SNPs (*r*^2^ < 0.01).

### 2.3. Genetic Associations with IHD and Ischemic Stroke

Genetic associations with IHD (*n*_case_ = 60,801, *n*_control_ = 123,504) in people mainly of European descent (77%) adjusted for genomic control and with ischemic stroke (*n*_case_ = 34,217, *n*_control_= 406,111) in people of European descent only adjusted for age, sex, study-specific covariates, and genomic control were obtained from summary statistics of CARDIoGRAMplusC4D 1000 Genomes [41] and MEGASTROKE consortium [42], respectively. Genetic associations with IHD and ischemic stroke, with diagnosis based on the International Classification of Diseases (ICD)-9 and/or 10 codes (Table S3), were also obtained from the UK Biobank [25] pan-ancestry summary statistics (assessed 18 January 2021) including 420,531 people of European ancestry adjusted for age, sex, age ∗ sex, age^2^, age^2^ ∗ sex, and the top 10 principal components. The participants in UK Biobank have no overlap with CARDIoGRAMplusC4D 1000 Genomes or MEGASTROKE.

### 2.4. Statistical Analysis

We aligned the exposures and outcomes using the effect allele across studies and also using effect allele frequency for palindromic SNPs. We used the F-statistic to evaluate instrument strength obtained from an approximation [43] and excluded SNPs with an F-statistic < 10, which indicates weak instruments. SNPs not available for the outcome were replaced by a highly correlated proxy (*r*^2^ ≥ 0.8) using *LDlink* in the appropriate population. Univariable inverse variance weighting (IVW) estimates with multiplicative random effects [44] accounting for correlations, obtained using the MR-Base “ld_matrix” function were used to assess associations of genetically predicted AA synthesis with lipid profile, blood pressure, adiposity, and measures of inflammation and coagulation overall and by sex if available. In sensitivity analysis, the weighted median, valid if >50% of information is from valid SNPs [45], MR-Egger, valid if instrument strength is independent of the direct effect [43], and leave-one-out analysis were used. We assessed sex differences using a two-sided z-test. We similarly assessed associations of these factors with AA synthesis. We used multivariable IVW accounting for correlations to assess associations of genetically predicted AA synthesis with IHD and ischemic stroke adjusted for the most likely mediators. Specifically, we pooled the SNPs for AA synthesis and a potential mediator, removed correlated SNPs (*r*^2^ < 0.01), extracted their associations with each exposure and outcome, and fitted a multivariable model. For consistency with the presentation of the mediators, we used one standard deviation (1.96% of total fatty acid) [46] as the unit of AA synthesis. We meta-analyzed estimates from consortia and the UK Biobank for IHD and ischemic stroke. We assessed multivariable instrument strength using the Sanderson-Windmejier multivariable conditional F-statistic, and assessed pleiotropy from the Q-statistic using the WSpiller/MVMR package [47]. We also used multivariable MR-Egger (orientated on AA synthesis) in multivariable models [48]. A non-zero intercept (*p* < 0.05) in MR-Egger indicates directional pleiotropy (some SNPs act beyond AA synthesis).

We calculated the effect sizes that can be detected with 80% power at a significance level of 0.05 for each CVD risk factor [49]. A Bonferroni-corrected significance level of 0.05/5 = 0.01 (given five classes of CVD risk factors) was considered for associations in univariable MR analysis. Analyses were conducted using R version 4.0.3 (The R Foundation for Statistical Computing, Vienna, Austria) and the R packages “MendelianRandomization”, “MVMR” and “metafor”. We used only publicly available summary statistics, which requires no ethical approval.

## 3. Results

We obtained eight SNPs (*p* < 5 × 10^−8^) predicting plasma phospholipid AA (i.e., AA synthesis), independent of rs174547 (*r*^2^ < 0.01) (Table S4). Three SNPs (rs2903922, rs760306, and rs472031) were associated with linoleic acid and dihomo-γ-linolenic acid (*p* < 5 × 10^−8^) but the associations were weaker than for AA synthesis; another SNP (rs1741) had a stronger association with dihomo-γ-linolenic acid and linoleic acid than with AA synthesis, while none of the eight SNPs were related to omega-3 PUFAs. Two SNPs (rs1741 and rs472031) were associated with HDL-C; one SNP (rs1741) was associated with TG and body composition, which were excluded in the analyses for HDL-C, TG, and adiposity accordingly. None of these eight SNPs was associated with key confounders (Table S5). The eight SNPs each had an F-statistic > 10 and collectively explained 5.3% of the variance in AA synthesis, while rs174547 explained 32% of the variance in AA synthesis [8] (Table S4). Two SNPs (rs259874 and rs472031) were not available for coagulation and had no proxy, while all of the SNPs were available for other outcomes. We had 80% power to detect an effect size ranging from 0.015 (BMI) to 0.212 (IL-6R) based on the eight SNPs and an effect size ranging from 0.006 (BMI) to 0.086 (IL-6R) based on rs174547 (Table S6). The number of proxies used for the published genetic variants for CVD risk factors and genetic associations are summarized in Tables S2 and S7, respectively. The SNPs predicting each CVD risk factor had a mean F-statistic > 10. We excluded rs174566 for TG, HDL-C and ApoA-I and rs174564 for LDL-C and ApoB in univariable and multivariable MR because they were associated with AA synthesis (*p* < 5 × 10^−8^) and strongly correlated with rs174547 (*r*^2^ = 0.95 and 0.97, respectively). A flowchart depicting the study is given in Figure S1.

**Figure 1:**
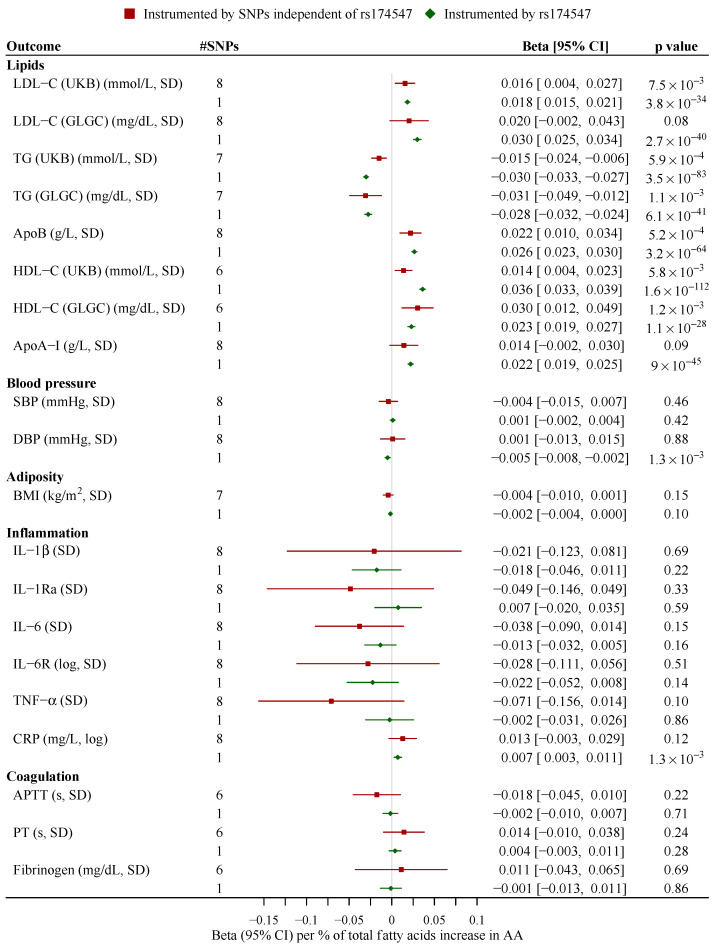
Associations of genetically predicted arachidonic acid synthesis with lipid profile, blood pressure, adiposity, and markers of inflammation and coagulation. The estimates based on SNPs independent of rs174547 were derived from inverse variance weighting with multiplicative random effects, while the estimates based on rs174547 were the Wald estimate. ApoA-I, apolipoprotein A-I; ApoB, apolipoprotein B; APTT, activated partial thromboplastin time; BMI, body mass index; CI, confidence interval; CRP, C-reactive protein; DBP, diastolic blood pressure; GLGC, the Global Lipids Genetics Consortium; HDL-C, high-density lipoprotein cholesterol; IL-6, interleukin-6; IL-6R, IL-6 receptor; LDL-C, low-density lipoprotein cholesterol; PT, prothrombin time; SBP, systolic blood pressure; SNP, single-nucleotide polymorphisms; TG, triglycerides; TNF-α, tumor necrosis factor-α; UKB, UK Biobank.

**Figure 2:**
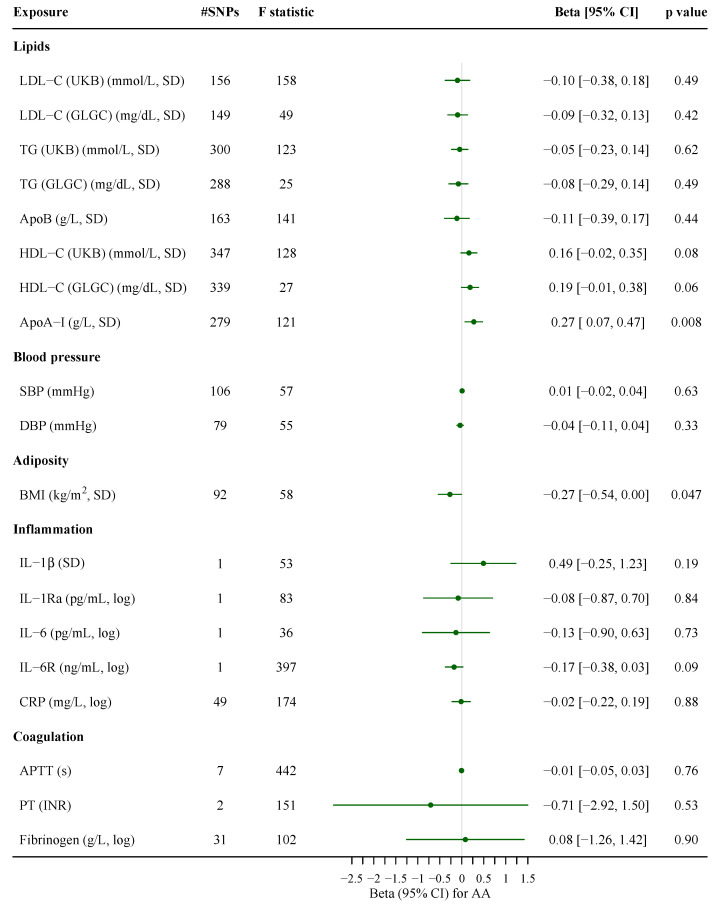
Associations of genetically predicted lipid profile, blood pressure, adiposity, and markers of inflammation and coagulation with arachidonic acid synthesis. The estimates based on two or more SNPs were derived from inverse variance weighting with multiplicative random effects, while the estimates based on one SNP were the Wald estimate. ApoA-I, apolipoprotein A-I; ApoB, apolipoprotein B; APTT, activated partial thromboplastin time; BMI, body mass index; CI, confidence interval; CRP, C-reactive protein; DBP, diastolic blood pressure; GLGC, the Global Lipids Genetics Consortium; HDL-C, high-density lipoprotein cholesterol; IL-6, interleukin-6; IL-6R, IL-6 receptor; INR, international normalized ratio; LDL-C, low-density lipoprotein cholesterol; PT, prothrombin time; SBP, systolic blood pressure; SNP, single-nucleotide polymorphisms; TG, triglycerides; UKB, UK Biobank.

Based on SNPs independent of rs174547, genetically higher AA synthesis was associated with higher ApoB, HDL-C and LDL-C (UKB) but lower TG. AA synthesis was not related to blood pressure, adiposity or measures of inflammation and coagulation (Figure 1). Leave-one-out analysis suggested no obvious outliers (Figure S2). Repeating the analysis excluding rs1741 gave similar estimates (Figure S3). Patterns were generally similar using rs174547 to predict AA; additional associations of AA with lower DBP and higher CRP were found (Figure 1). The weighted median showed similar estimates. The MR-Egger intercept indicated no horizontal pleiotropy (Table S8). The associations of AA with lipid profile, blood pressure and CRP did not differ by sex, with a stronger negative association with BMI for women than men (*p* = 0.02 for sex difference) based on rs174547 (Figure S4). Genetically predicted lipid profile, blood pressure, adiposity, and markers of inflammation and coagulation were not associated with AA synthesis, except for a positive association of ApoA-I and a nominally negative association of BMI with AA synthesis (Figure 2).

**Figure 3:**
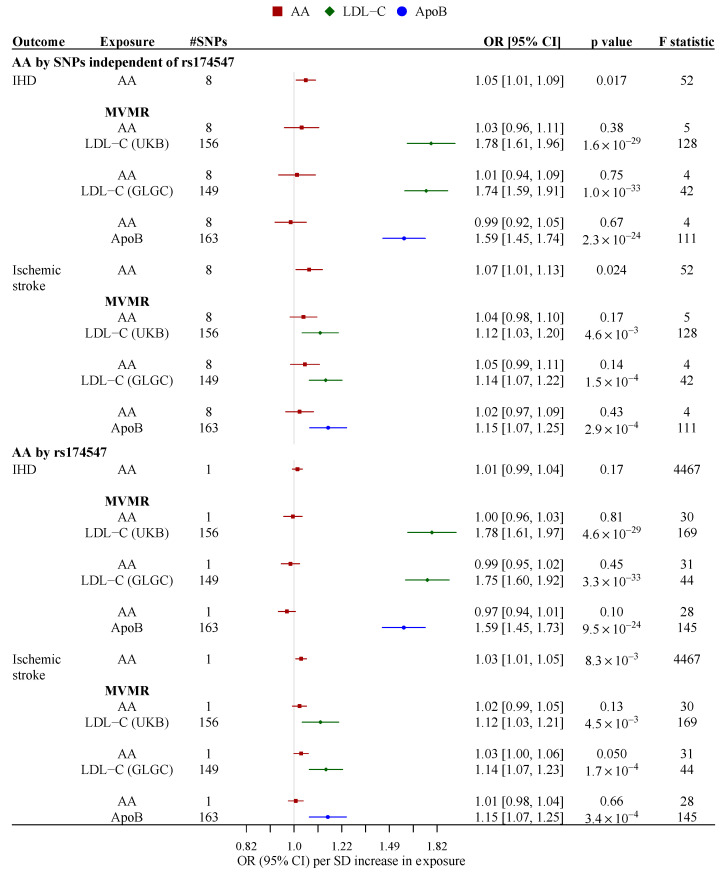
Multivariable Mendelian randomization estimates for associations of genetically predicted arachidonic acid synthesis with ischemic heart disease and ischemic stroke after adjustment for LDL-C or ApoB. F statistic indicated mean F-statistic in the univariable models and conditional F-statistic in the multivariable models. For IHD, the estimates were derived from meta-analyses of random-effect multivariable inverse variance weighting estimates from CARDIoGRAMplusC4D 1000 Genomes (*n*_case_ = 60,801, *n*_control_ = 123,504) and UK Biobank (*n*_case_ = 37,672, *n*_control_ = 382,052); for ischemic stroke, the estimates were derived from meta-analyses of random-effect multivariable inverse variance weighting estimates from MEGASTROKE (*n*_case_ = 34,217, *n*_control_ = 406,111) and UK Biobank (*n*_case_ = 4275, *n*_control_ = 416,256). For all meta-analyzed estimates, *p* for heterogeneity >0.05. AA, arachidonic acid; ApoB, apolipoprotein B; CI, confidence interval; GLGC, the Global Lipids Genetics Consortium; IHD, ischemic heart disease; LDL-C, low-density lipoprotein cholesterol; MVMR, multivariable mendelian randomization; SNP, single-nucleotide polymorphisms; UKB, UK Biobank.

In multivariable MR, we assessed the association of AA synthesis with IHD and ischemic stroke adjusting for LDL-C or ApoB, but not both because they are highly correlated. We did not consider other lipids as mediators because AA decreased TG while HDL-C and ApoA-I are not thought to have causal roles in CVD [50,51]. The conditional F-statistics for AA synthesis based on SNPs independent of rs174547 were 4.6 and 4.3 in the multivariable models adjusted for LDL-C and ApoB, respectively (Figure 3). The Q-statistics for instrument pleiotropy were significant (*p* < 0.05) but the multivariable MR-Egger intercepts were not (Table S9). Using SNPs independent of rs174547, the associaion of genetically predicted AA synthesis with IHD was attenuated to the null after adjusting for ApoB or LDL-C. The estimate for ischemic stroke was partly attenuated after adjusting for ApoB or LDL-C (Figure 3). The patterns were similar using rs174547 to predict AA synthesis.

## 4. Discussion

Genetically predicted AA synthesis was associated with higher ApoB, HDL-C and possibly LDL-C and lower TG, but not with blood pressure, adiposity, markers of inflammation and coagulation with no obvious sex difference. Genetically predicted lipid profile, blood pressure, adiposity, and markers of inflammation and coagulation were not associated with AA synthesis. The association of AA synthesis with IHD was attenuated to the null and the association of AA synthesis with ischemic stroke was partly attenuated after adjusting for ApoB or LDL-C.

Our findings of the associations of AA synthesis with higher HDL-C and possibly LDL-C and lower TG are consistent with a previous MR [10] and observational studies [11,52]. We additionally found a positive association of AA synthesis with ApoB, which has not been previously investigated using MR. Several small trials have shown little effect of dietary or supplemental AA on blood lipids [13,14,15]. A possible reason for the discrepancy is that MR assesses lifelong effects while the trials assessed the short-term effect of AA intake (< 8 weeks). Null findings from trials could also be due to low power given the small number of subjects (*n* = 10–24). Genetically predicted lipid profile was not associated with AA synthesis, suggesting that AA synthesis affects lipids, specifically ApoB and LDL-c, but not the reverse. ApoB is higher in men than women [53], but whether AA affects sex hormones and thereby ApoB and IHD has not been investigated. In this study, we found no obvious difference by sex for the association of AA synthesis with ApoB. An in vitro experimental study showed that AA administration increased ApoB secretion by 171% in human hepatoma cells [54].

The lack of association of AA synthesis with blood pressure is inconsistent with a prior MR study [10] suggesting an inverse association with DBP. However, our estimates are more independent of the highly pleiotropic SNP underlying the Flores and Inuit haplotype. Our findings are more consistent with RCTs showing no such effect of supplemental AA [14,16]. As far as we know, this is the first MR study examining the associations of AA synthesis with adiposity and markers of inflammation and coagulation. Consistent with findings from RCTs, no association of AA synthesis with adiposity was found [14,17]. Our finding of no significant associations with selected inflammatory markers is consistent with some RCTs of AA supplementation showing little effect [13,16,55] but not with another trial showing reduced IL-6 [17]. However, given the limited sample size (*n* = 8293) of the cytokines GWAS [28], an association of AA synthesis with circulating IL-6 remains possible, which, as reduced IL-6R signaling, is associated with lower IHD risk [37]. The lack of associations of AA synthesis with measures of coagulation is consistent with some trials [14,16,20] but not with others using higher doses (≥2 g/d) of AA [18,19]. We cannot exclude that 2 g/d or higher intake of AA, or a corresponding increase in plasma AA (about 7% of total fatty acids) [6], may affect coagulation or platelet aggregation. These results should be interpreted with caution because genetic associations of AA synthesis with APTT and PT are from populations of different ancestries. Future investigations using European-specific genetic associations for a comprehensive set of coagulation factors is warranted. Multivariable MR suggested that the association of AA with IHD was attenuated to the null after accounting for ApoB or LDL-C. Recent MR suggested that ApoB, rather than LDL-C, is the key lipid trait in the etiology of IHD and ischemic stroke [22,23].

Limitations of the study exist. Firstly, MR should fulfil three assumptions, i.e., relevance, independence and exclusion restriction. We used genetic variants predicting AA synthesis at genome-wide significance with F-statistics > 10 in univariable MR. We used a less stringent linkage disequilibrium threshold (*r*^2^ < 0.01) to obtain independent variants for AA synthesis, which may be slightly correlated. However, the univariable and multivariable MR analyses accounted for linkage disequilibrium using a correlation matrix. The conditional F-statistics for AA synthesis in multivariable MR were calculated without considering the covariance of AA synthesis and LDL-C or ApoB because they are from separate samples [47]. The conditional F-statistics for AA synthesis were quite low, so bias from a weak instrument is possible, which could be in either direction [47]. The GWAS for AA synthesis and some outcomes overlap slightly (~4.2%), which is less likely to create population stratification bias. The genetic variants for AA synthesis were unrelated to key confounders such as socio-economic position and lifestyle. We specifically used genetic variants independent of the highly pleiotropic Flores and Inuit haplotype rs174547 [21] and excluded variants associated with known pleiotropic traits. None of the variants for AA synthesis were related to any omega-3 PUFA, including EPA and DHA, which may affect CVD risk factors. Sensitivity analysis by excluding any variant related to other omega-6 PUFAs gave similar results, which, however, may not sufficiently remove the effects of other omega-6 PUFAs. The MR-Egger intercepts indicated no unknown pleiotropy. Leave-one-out analysis suggested no obvious outliers. Secondly, the effect of endogenous AA synthesis may not necessarily reflect that of exogenous AA, although plasma AA relates to intake of AA (82–3600 mg/day) dose-dependently [6]. Rather, the observed effect of plasma phospholipid AA may partly reflect the effect of AA or omega-6 PUFA synthesis. Thirdly, the AA synthesis GWAS is less densely genotyped, leaving nearly 30% of instruments for lipid profile and blood pressure not available with no proxy. However, the instruments for all CVD risk factors had mean F-statistics > 10, lowering the possibility of weak instrument bias. Fourthly, most GWAS concern people of European ancestry only, although CARDIoGRAMplusC4D 1000 Genomes has 23% of non-European ancestry, which could result in bias from population stratification, and Biobank Japan is of Japanese ancestry. However, CARDIoGRAMplusC4D 1000 Genomes used genomic control [41] and the results for IHD were similar to those from the UK Biobank. Nevertheless, the SNPs predicting AA synthesis from people of European descent might not apply to the Japanese population. GWAS of coagulation factors from different populations would be very helpful. Fifth, we did not consider type 2 diabetes as a potential mediator because previous MR suggested no association of AA synthesis with it after excluding the Flores and Inuit haplotype represented by rs174547 [56]. Sixth, the findings might not generalize to non-Europeans or to vegetarians; causes would be expected to act consistently, although relevance may vary by setting [57]. Seventh, selection bias could be an issue for ischemic stroke, which typically occurs later than IHD, which may bias the estimates towards the null [58]. Eighth, we were limited by the availability of large GWAS or markers of inflammation and coagulation, so we cannot exclude the effects of AA synthesis on key inflammatory pathways or elements in the coagulation cascade. Finally, we cannot determine whether ApoB or LDL-C is more likely to mediate the association of AA synthesis with IHD, which needs further investigation. However, other evidence suggests ApoB rather than LDL-C is the key lipid in IHD and stroke [22,23].

## 5. Conclusions

Our study found associations of genetically predicted AA synthesis with higher ApoB, HDL-C and possibly LDL-C and lower TG, but not with blood pressure, adiposity, selected inflammatory factors or available measures of coagulation with no obvious sex differences. Whether AA synthesis affects coagulation in Western populations needs further investigation. The association of AA synthesis with IHD and ischemic stroke may be mediated by ApoB or LDL-C, but most likely by ApoB. Our study provides new insights concerning underlying mechanisms by which AA synthesis may affect IHD and ischemic stroke.

## Data Availability

The data presented in this study are available in Supplementary Materials.

## Supplementary Materials

The following are available online at https://www.mdpi.com/article/10.3390/nu13051489/s1, Table S1: Data sources of lipid profile, blood pressure, adiposity, and markers of inflammation and coagulation overall and by sex. Table S2: Genetic instruments for lipid profile, blood pressure, adiposity, and markers of inflammation and coagulation. Table S3: Data sources and ICD codes of IHD and ischemic stroke. Table S4: The genetic variants predicting AA synthesis reflected by plasma phospholipid AA and genetic associations with outcomes. Table S5: Relations of the SNPs predicting AA synthesis with potential confounders and known related traits of the SNPs at genome-wide significance. Table S6: Effect sizes under 80% power for the associations of genetically predicted AA synthesis with CVD risk factors. Table S7: The genetic variants predicting lipids, blood pressure, adiposity, and markers of inflammation and coagulation. Figure S1: Flowchart of the univariable and multivariable Mendelian randomization. Figure S2: Leave-one-out analyses of the associations of genetically predicted AA synthesis with lipid profile, blood pressure, adiposity, and markers of inflammation and coagulation. Figure S3: Associations of genetically predicted AA synthesis with lipid profile, blood pressure, adiposity, and markers of inflammation and coagulation after excluding rs1741. Figure S4: Associations of genetically predicted AA synthesis with lipid profile, blood pressure, BMI, and CRP by sex in the UK Biobank. Table S8: Associations of genetically predicted AA synthesis with lipid profile, blood pressure, adiposity, and markers of inflammation and coagulation using all methods. Table S9: Multivariable Mendelian randomization estimates for associations of genetically predicted AA synthesis with IHD and ischemic stroke after adjustment for LDL-C or ApoB using all methods.

## List of Abbreviations

**Abbreviation**	**Full Name**
AA	arachidonic acid
ApoA-I	apolipoprotein A-I
ApoB	apolipoprotein B
APTT	activated partial thromboplastin time
BBJ	the Biobank Japan
BMI	body mass index
CHARGE	Cohorts for Heart and Aging Research in Genomic Epidemiology
CI	confidence interval
CIWG	the Cohorts for Heart and Aging Research in Genomic Epidemiology Inflammation Working Group
CRP	C-reactive protein
CVD	cardiovascular disease
GWAS	genome-wide association study
DBP	diastolic blood pressure
DHA	docosahexaenoic acid
EPA	eicosapentaenoic acid
GIANT	the Genetic Investigation of ANthropometric Traits Consortium
GLGC	the Global Lipids Genetics Consortium
HDL-C	high-density lipoprotein cholesterol
IHD	ischemic heart disease
IL-1β	interleukin-1 β
IL-1Ra	interleukin-1 receptor antagonist
IL-6	interleukin-6
IL-6R	IL-6 receptor
IVW	inverse variance-weighted
LDL-C	low-density lipoprotein cholesterol
MR	Mendelian randomization
OR	odds ratio
PT	prothrombin time
PUFA	polyunsaturated fatty acid
RCT	randomized controlled trial
SBP	systolic blood pressure
SD	standard deviation
SNP	single-nucleotide polymorphisms
TG	triglycerides
TNF-α	tumor necrosis factor-α
UKB	UK Biobank
