# Supplementary material for: Associations of Arachidonic Acid Synthesis with Cardiovascular Risk Factors and Relation to Ischemic Heart Disease and Stroke: A Univariable and Multivariable Mendelian Randomization Study
Source: Nutrients. 2021 Apr 28;13(5):1489. doi: 10.3390/nu13051489 (PMC8146807; doi:10.3390/nu13051489)
Supplement: Supplementary file 1 [file nutrients-13-01489-s001.zip › nutrients-1177710-supplementary/Figure S1-S4.pdf]

Supplementary Materials

for

Associations of arachidonic acid synthesis with cardiovascular risk factors and relation to ischemic heart disease and stroke: a univariable and multivariable Mendelian Randomization study

Ting Zhang[1], Shiu Lun Au Yeung[1], C. Mary Schooling[1, 2]

1. School of Public Health, Li Ka Shing Faculty of Medicine, The University of Hong Kong, Hong Kong SAR, China

2. School of Public Health and Health Policy, City University of New York, New York, USA

Corresponding Author

C. Mary Schooling

E-mail: cms1@hku.hk

Supplementary Figures

Figure S1 Flowchart of the univariable and multivariable Mendelian randomization.

Figure S2 Leave-one-out analyses of the associations of genetically predicted AA synthesis with lipid profile, blood pressure, adiposity, and markers of inflammation and coagulation.

Figure S3 Associations of genetically predicted AA synthesis with lipid profile, blood pressure, adiposity, and markers of inflammation and coagulation after excluding rs1741.

Figure S4 Associations of genetically predicted AA synthesis with lipid profile, blood pressure, BMI, and CRP by sex in the UK Biobank.

Figure S1 Flowchart of the univariable and multivariable Mendelian randomization.

AA synthesis reflected by plasma phospholipid AA from CHARGE (*n*=8,631)

- **Univariable MR: associations of AA with CVD risk factors:**
- Instruments for AA: 8 uncorrelated (r^2<0.01) SNPs, independent of rs174547, predicting AA ($P<5\times10^{-8}$), and rs174547, separately.
- Removing pleiotropic SNPs: rs1741 and rs472031 for HDL-C; rs1741 for TG and BMI;
- Unavailable without proxy: rs259874 and rs472031) were not available for APTT, PT and fibrinogen.

- **Univariable MR: associations of CVD risk factors with AA:**
- Instruments for CVD risk factors: LDL-C (220), TG (440), ApoB (255), HDL-C (534), ApoA-I (440), SBP (130), DBP (91), BMI (97), IL-1β (1), IL-1ra (2), IL-6 (3), IL-6R (1), CRP (52), APTT (8) and PT (2), fibrinogen (41).
- Removing pleiotropic SNPs: rs174566 for TG, HDL-C and ApoA-I and rs174564 for LDL-C and ApoB.

Genetic associations with CVD risk factors:
- Overall:
- Lipid profile: LDL-C, TG, ApoB, HDL-C, ApoA-I from the UK Biobank (*n*=420,531, European ancestry only); and LDL-C, TG, HDL-C from GLGC (*n*=188,578, European ancestry only).
- Blood pressure: SBP, DBP from the UK Biobank (*n*=420,531, European ancestry only).
- Adiposity: BMI from a meta-analysis of GIANT and UK Biobank (*n*=681,275, European ancestry).
- Inflammation: IL-1β, IL-1Ra, IL-6, TNF-α from Ahola-Olli et al (*n*=8,293, Finnish ancestry), IL-6R from the INTERVAL study (*n*=3,301, European ancestry), CRP from CIWG (*n*=204,402, European ancestry).
- Coagulation: APTT, PT, fibrinogen from BBJ (*n*=58,110, Japanese ancestry).
- Sex-specific:
- Lipid profile, blood pressure, BMI, CRP: the UK Biobank (167,020 white British men and 194,174 women)

Genetic instruments for CVD risk factors:
- Lipid profile: LDL-C (220), TG (440), ApoB (255), HDL-C (534), ApoA-I (440) from the UK Biobank (*n*=441,016, European ancestry); association data also obtained from GLGC.
- Blood pressure: SBP (130), DBP (91) from a meta-analysis of ICBP and the UK Biobank (*n*=757,601, European ancestry only).
- Adiposity: BMI (97) from a meta-analysis of GIANT and UK Biobank (*n*=339,224, mainly European ancestry).
- Inflammation: IL-1β (1) (Sliz et al), IL-1Ra (2) (Herder et al), IL-6 (3) (IL6R MR Consortium), IL-6R (1) (Sarwar et al), CRP (52) (CIWG).
- Coagulation: APTT (8) and PT (2) (Tang et al), fibrinogen (41) (CHARGE).

- **Multivariable MR: instruments for AA**
- 8 SNPs independent of rs174547
- rs174547

- **Multivariable MR: instruments for LDL-C and ApoB**
- 220 SNPs for LDL-C, 255 SNPs for ApoB (association data from the UK Biobank (*n*=420,531) and GLGC (LDL-C only)).
- Removing pleiotropic SNPs: rs174564 for LDL-C and ApoB.

- **Multivariable MR: associations of AA with IHD and ischemic stroke mediated by LDL-C or ApoB:**

Genetic associations with IHD and ischemic stroke:
- IHD: CARDIoGRAMplusC4D 1000 Genomes (cases=60,801, controls=123,504) and the UK Biobank (cases=37,672, controls=382,052) (separately and meta-analyzed).
- Ischemic stroke: MEGASTROKE (cases=34,217, controls=406,111) and the UK Biobank (cases=4,275, controls=416,256).

ApoA-I, apolipoprotein A-I; ApoB, apolipoprotein B; APTT, activated partial thromboplastin time; BBJ, the Biobank Japan; BMI, body mass index; CI, confidence interval; CRP, C-reactive protein; DBP, diastolic blood pressure; GIANT, the Genetic Investigation of ANthropometric Traits Consortium; GLGC, the Global Lipids Genetics Consortium; HDL-C, high-density lipoprotein cholesterol; ICBP, the International Consortium of Blood Pressure-Genome Wide Association Studies; LDL-C, low-density lipoprotein cholesterol; PT, prothrombin time; SBP, systolic blood pressure; SNP, single-nucleotide polymorphisms; TG, triglycerides.

Figure S2 Leave-one-out analyses of the associations of genetically predicted arachidonic acid synthesis with lipid profile, blood pressure, adiposity, and markers of inflammation and coagulation.

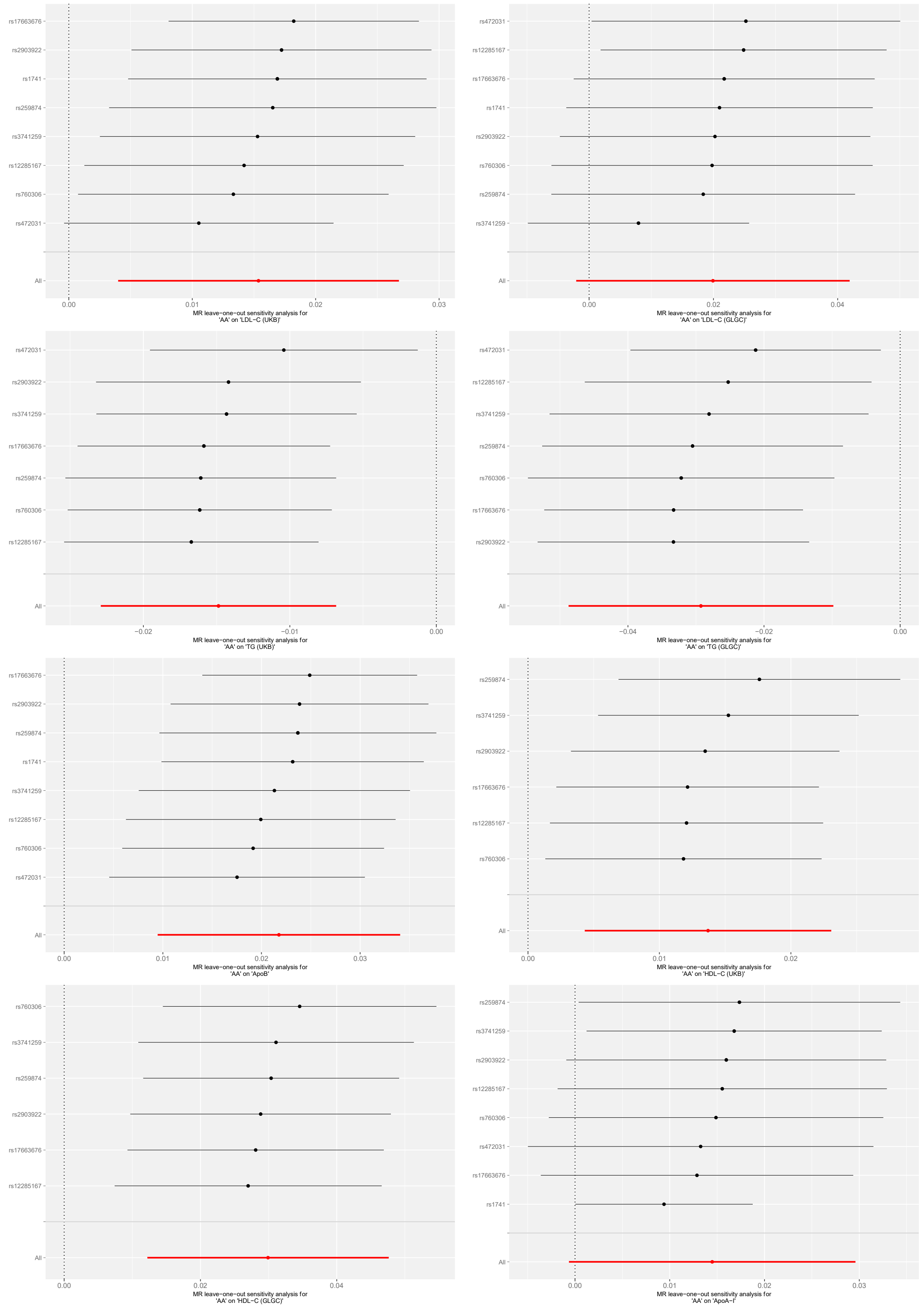

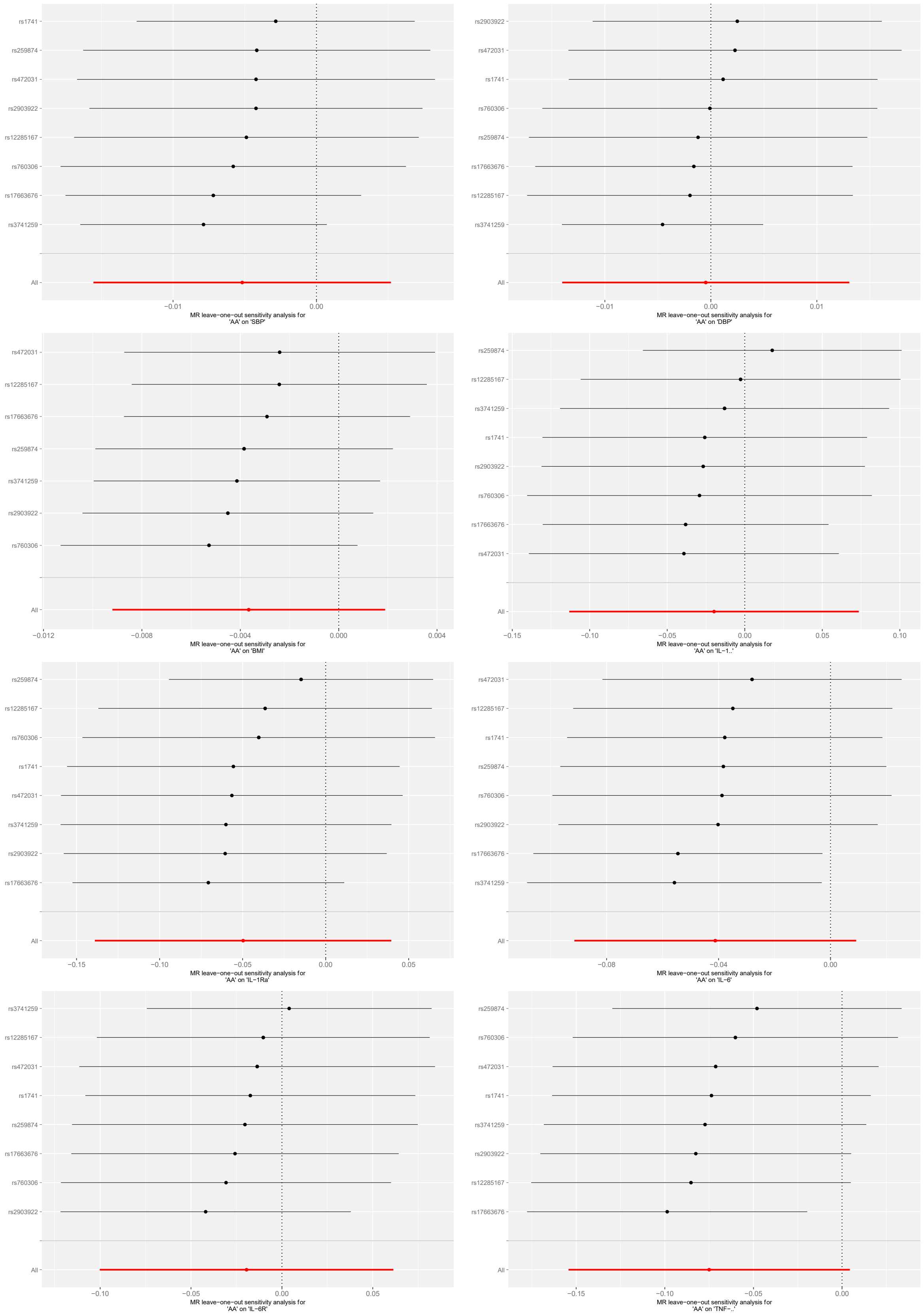
rs1741
rs259874
rs472031
rs2903922
rs12285167
rs760306
rs17663676
rs3741259
All
−0.01
0.00
MR leave−one−out sensitivity analysis for
'AA' on 'SBP'
rs2903922
rs472031
rs1741
rs760306
rs259874
rs17663676
rs12285167
rs3741259
All
−0.01
0.00
0.01
MR leave−one−out sensitivity analysis for
'AA' on 'DBP'
rs472031
rs12285167
rs17663676
rs259874
rs3741259
rs2903922
rs760306
All
−0.012
−0.008
−0.004
0.000
0.004
MR leave−one−out sensitivity analysis for
'AA' on 'BMI'
rs259874
rs12285167
rs3741259
rs1741
rs2903922
rs760306
rs17663676
rs472031
All
−0.15
−0.10
−0.05
0.00
0.05
0.10
MR leave−one−out sensitivity analysis for
'AA' on 'IL−1..'
rs259874
rs12285167
rs760306
rs1741
rs472031
rs3741259
rs2903922
rs17663676
All
−0.15
−0.10
−0.05
0.00
0.05
MR leave−one−out sensitivity analysis for
'AA' on 'IL−1Ra'
rs472031
rs12285167
rs1741
rs259874
rs760306
rs2903922
rs17663676
rs3741259
All
−0.08
−0.04
0.00
MR leave−one−out sensitivity analysis for
'AA' on 'IL−6'
rs3741259
rs12285167
rs472031
rs1741
rs259874
rs17663676
rs760306
rs2903922
All
−0.10
−0.05
0.00
0.05
MR leave−one−out sensitivity analysis for
'AA' on 'IL−6R'
rs259874
rs760306
rs472031
rs1741
rs3741259
rs2903922
rs12285167
rs17663676
All
−0.15
−0.10
−0.05
0.00
MR leave−one−out sensitivity analysis for
'AA' on 'TNF−..'

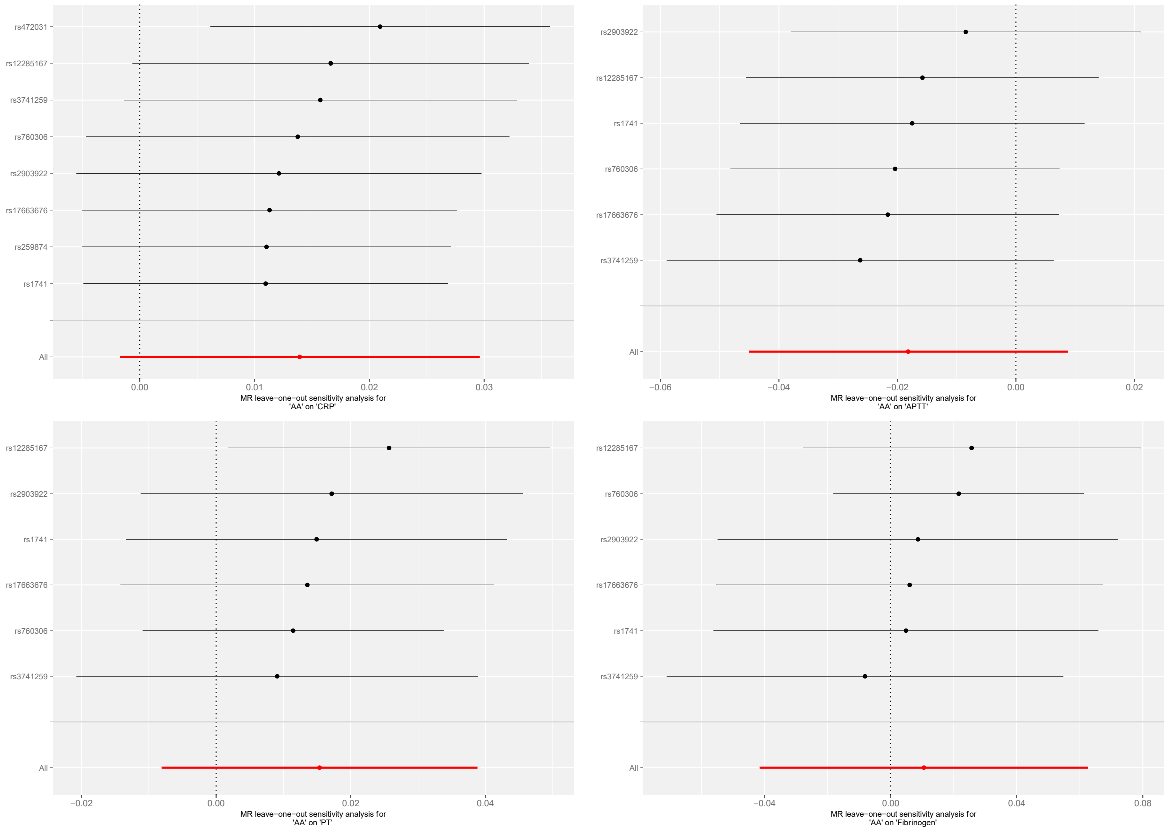

ApoA-I, apolipoprotein A-I; ApoB, apolipoprotein B; APTT, activated partial thromboplastin time; BMI, body mass index; CRP, C-reactive protein; DBP, diastolic blood pressure; GLGC, the Global Lipids Genetics Consortium; HDL-C, high-density lipoprotein cholesterol; LDL-C, low-density lipoprotein cholesterol; PT, prothrombin time; SBP, systolic blood pressure; TG, triglycerides; UKB, UK Biobank.

Figure S3 Associations of genetically predicted AA synthesis with lipid profile, blood pressure, adiposity, and markers of inflammation and coagulation after excluding rs1741.

Outcome	SNPs	Beta [95% CI]	P value
**Lipids**			
LDL−C (UKB) (mmol/L, SD)	7	0.017 [0.005, 0.029]	4.8×10^{-3}
LDL−C (GLGC) (mg/dL, SD)	7	0.021 [−0.003, 0.046]	0.09
TG (UKB) (mmol/L, SD)	7	−0.015 [−0.024, −0.006]	5.9×10^{-4}
TG (GLGC) (mg/dL, SD)	7	−0.031 [−0.049, −0.012]	1.1×10^{-3}
ApoB (g/L, SD)	7	0.023 [0.010, 0.036]	4.9×10^{-4}
HDL−C (UKB) (mmol/L, SD)	6	0.014 [0.004, 0.023]	5.8×10^{-4}
HDL−C (GLGC) (mg/dL, SD)	6	0.030 [0.012, 0.049]	1.2×10^{-3}
ApoA−I (g/L, SD)	7	0.010 [0.000, 0.020]	0.05
**Blood pressure**			
SBP (mmHg, SD)	7	−0.002 [−0.012, 0.008]	0.69
DBP (mmHg, SD)	7	0.003 [−0.012, 0.018]	0.72
**Adiposity**			
BMI (kg/m^2, SD)	7	−0.004 [−0.010, 0.001]	0.15
**Inflammation**			
IL−1β (SD)	7	−0.027 [−0.139, 0.086]	0.64
IL−1Ra (SD)	7	−0.053 [−0.161, 0.054]	0.33
IL−6 (SD)	7	−0.035 [−0.089, 0.019]	0.21
IL−6R (log, SD)	7	−0.028 [−0.121, 0.065]	0.55
TNF−α (SD)	7	−0.070 [−0.165, 0.025]	0.15
CRP (mg/L, log)	7	0.010 [−0.005, 0.025]	0.20
**Coagulation**			
APTT (s, SD)	5	−0.017 [−0.047, 0.012]	0.26
PT (s, SD)	5	0.013 [−0.015, 0.042]	0.35
Fibrinogen (mg/dL, SD)	5	0.006 [−0.056, 0.068]	0.84

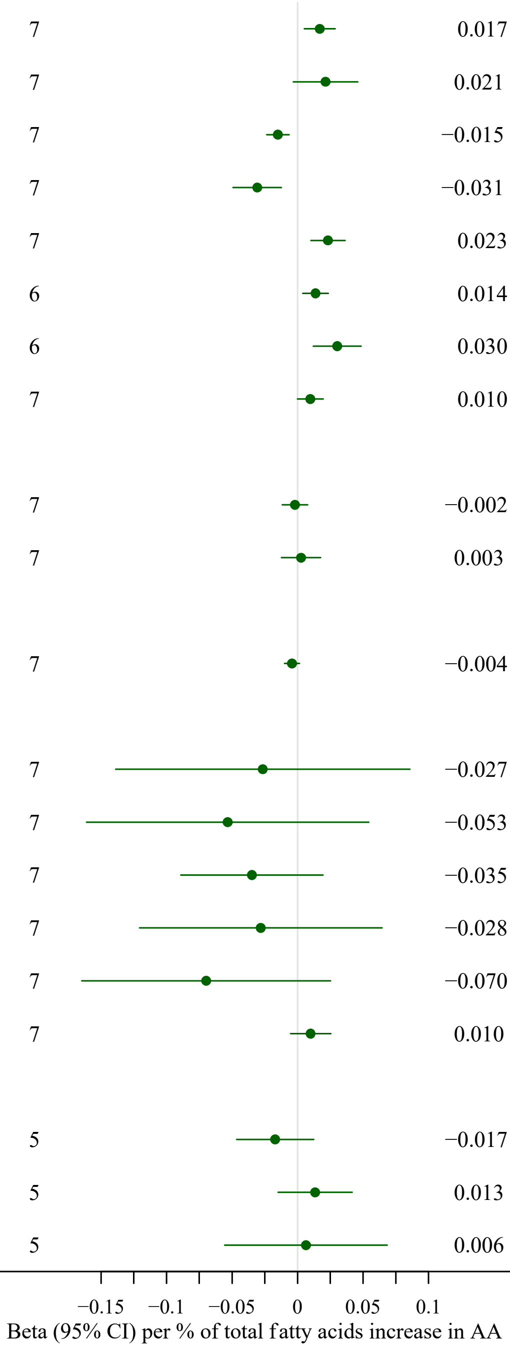

The estimates were derived from inverse variance weighting with multiplicative random effects. AA, arachidonic acid; ApoA-I, apolipoprotein A-I; ApoB, apolipoprotein B; APTT, activated partial thromboplastin time; BBJ, the Biobank Japan; BMI, body mass index; CI, confidence interval; CRP, C-reactive protein; DBP, diastolic blood pressure; GIANT, the Genetic Investigation of ANthropometric Traits Consortium; GLGC, the Global Lipids Genetics Consortium; HDL-C, high-density lipoprotein cholesterol; ICBP, the International Consortium of Blood Pressure-Genome Wide Association Studies; LDL-C, low-density lipoprotein cholesterol; PT, prothrombin time; SBP, systolic blood pressure; SNP, single-nucleotide polymorphisms; TG, triglycerides; UKB, UK Biobank.

Figure S4 Associations of genetically predicted AA synthesis with lipid profile, blood pressure, BMI, and CRP by sex in the UK Biobank.

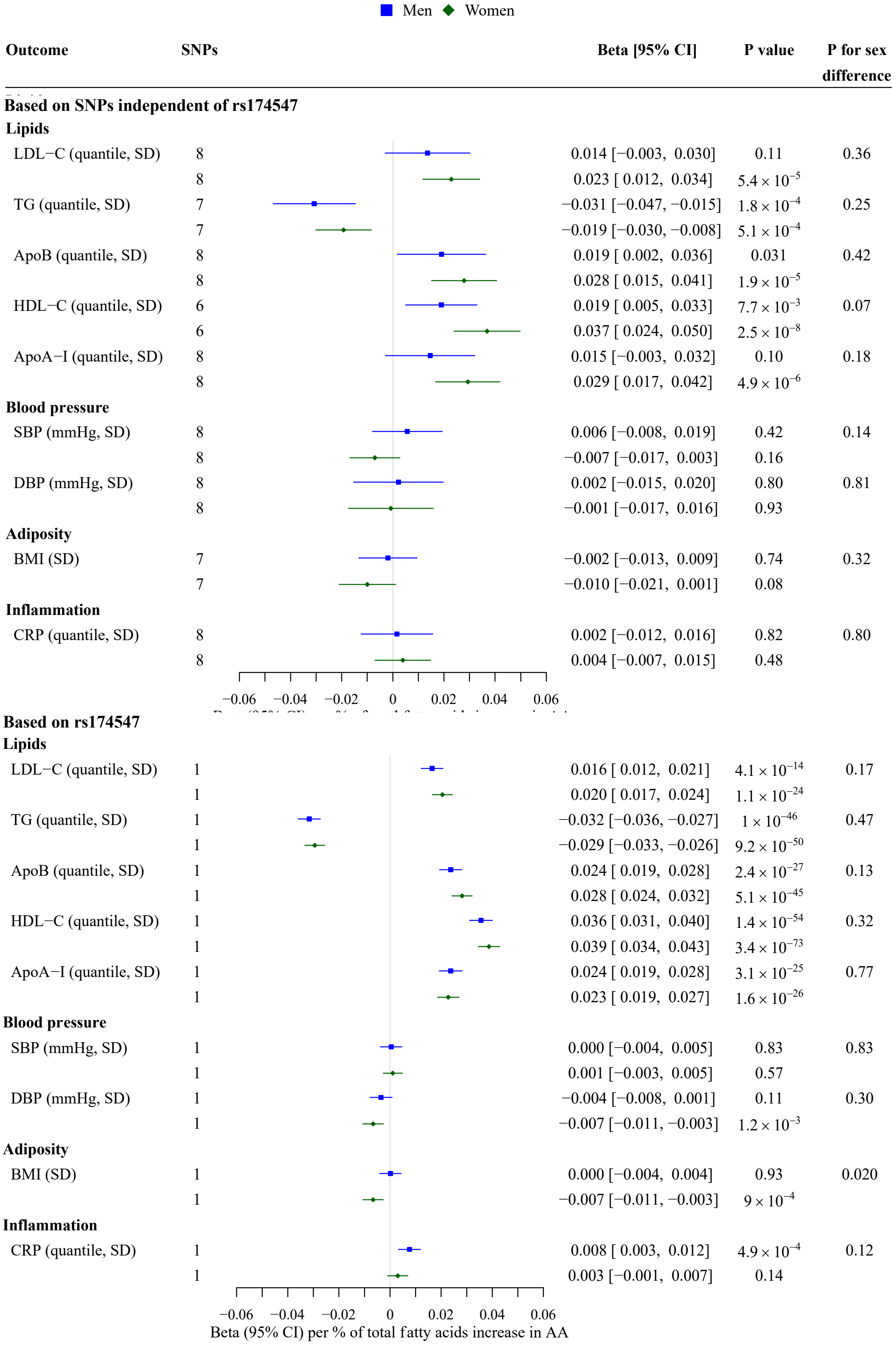

Outcome	SNPs	Beta [95% CI]	P value	P for sex difference
**Based on SNPs independent of rs174547**				
**Lipids**				
LDL−C (quantile, SD)	8	0.014 [−0.003, 0.030]	0.11	0.36
	8	0.023 [0.012, 0.034]	5.4×10^{-5}	
TG (quantile, SD)	7	−0.031 [−0.047, −0.015]	1.8×10^{-4}	0.25
	7	−0.019 [−0.030, −0.008]	5.1×10^{-4}	
ApoB (quantile, SD)	8	0.019 [0.002, 0.036]	0.031	0.42
	8	0.028 [0.015, 0.041]	1.9×10^{-5}	
HDL−C (quantile, SD)	6	0.019 [0.005, 0.033]	7.7×10^{-3}	0.07
	6	0.037 [0.024, 0.050]	2.5×10^{-8}	
ApoA−I (quantile, SD)	8	0.015 [−0.003, 0.032]	0.10	0.18
	8	0.029 [0.017, 0.042]	4.9×10^{-6}	
**Blood pressure**				
SBP (mmHg, SD)	8	0.006 [−0.008, 0.019]	0.42	0.14
	8	−0.007 [−0.017, 0.003]	0.16	
DBP (mmHg, SD)	8	0.002 [−0.015, 0.020]	0.80	0.81
	8	−0.001 [−0.017, 0.016]	0.93	
**Adiposity**				
BMI (SD)	7	−0.002 [−0.013, 0.009]	0.74	0.32
	7	−0.010 [−0.021, 0.001]	0.08	
**Inflammation**				
CRP (quantile, SD)	8	0.002 [−0.012, 0.016]	0.82	0.80
	8	0.004 [−0.007, 0.015]	0.48	
**Based on rs174547**				
**Lipids**				
LDL−C (quantile, SD)	1	0.016 [0.012, 0.021]	4.1×10^{-14}	0.17
	1	0.020 [0.017, 0.024]	1.1×10^{-24}	
TG (quantile, SD)	1	−0.032 [−0.036, −0.027]	1×10^{-46}	0.47
	1	−0.029 [−0.033, −0.026]	9.2×10^{-50}	
ApoB (quantile, SD)	1	0.024 [0.019, 0.028]	2.4×10^{-27}	0.13
	1	0.028 [0.024, 0.032]	5.1×10^{-45}	
HDL−C (quantile, SD)	1	0.036 [0.031, 0.040]	1.4×10^{-54}	0.32
	1	0.039 [0.034, 0.043]	3.4×10^{-73}	
ApoA−I (quantile, SD)	1	0.024 [0.019, 0.028]	3.1×10^{-25}	0.77
	1	0.023 [0.019, 0.027]	1.6×10^{-26}	
**Blood pressure**				
SBP (mmHg, SD)	1	0.000 [−0.004, 0.005]	0.83	0.83
	1	0.001 [−0.003, 0.005]	0.57	
DBP (mmHg, SD)	1	−0.004 [−0.008, 0.001]	0.11	0.30
	1	−0.007 [−0.011, −0.003]	1.2×10^{-3}	
**Adiposity**				
BMI (SD)	1	0.000 [−0.004, 0.004]	0.93	0.020
	1	−0.007 [−0.011, −0.003]	9×10^{-4}	
**Inflammation**				
CRP (quantile, SD)	1	0.008 [0.003, 0.012]	4.9×10^{-4}	0.12
	1	0.003 [−0.001, 0.007]	0.14	

The estimates based on SNPs independent of rs174547 were derived from inverse variance weighting with multiplicative random effects, while the estimates based on rs174547 were the Wald estimate. AA, arachidonic acid; ApoA-I, apolipoprotein A-I; ApoB, apolipoprotein B; BMI, body mass index; CI, confidence interval; CRP, C-reactive protein; DBP, diastolic blood pressure; HDL-C, high-density lipoprotein cholesterol; LDL-C, low-

density lipoprotein cholesterol; SBP, systolic blood pressure; SNP, single-nucleotide polymorphisms; TG, triglycerides.
